# Wnt/β-catenin pathway as a potential target for Parkinson’s disease: a cohort study of romosozumab using routinely collected health data in Japan

**DOI:** 10.3389/fphar.2024.1411285

**Published:** 2024-07-22

**Authors:** Shoichiro Inokuchi, Koji Shimamoto

**Affiliations:** Research and Analytics Department, Real World Data Co., Ltd., Kyoto, Japan

**Keywords:** osteoporosis, electronic health records, drug repositioning, Wnt signaling pathway, Parkinson disease

## Abstract

**Introduction:**

Romosozumab is a monoclonal antibody approved for osteoporosis which targets sclerostin, an endogenous inhibitor of Wnt/β-catenin pathway. Given the essential roles of the Wnt/β-catenin pathway in various tissues, we hypothesized romosozumab treatment may influence other conditions.

**Methods:**

This cohort study included patients prescribed romosozumab or parathyroid receptor (PTHR) agonists after 1 January 2019, using a Japanese electronic medical record database. The outcomes of interest included autoimmune disease, interstitial pneumonia, cardiovascular outcome, Alzheimer’s disease, Parkinson’s disease (PD), serious infections, and malignancies. A stabilized inverse probability-weighted Cox proportional hazard model was used to estimate the hazard ratios. Age- and gender-based subgroup analyses were conducted. Exploratory outcomes based on three-digit International Classification of Diseases 10th Revision-based were also examined.

**Results:**

In total, 2,673 patients treated with romosozumab and 5,980 treated with PTHR agonists were identified, respectively. While most outcomes of interest showed no association with romosozumab, the risk of PD decreased with romosozumab (hazard ratio [95% confidence interval], 0.37 [0.14–0.94]) compared with PTHR agonist. Regarding the cardiovascular outcome, no notable association was identified overall; however, gender-based subgroup analysis suggested that male sex may be a potential risk factor with romosozumab treatment. Only 16 of 903 exploratory outcomes were potentially influenced by romosozumab.

**Conclusion:**

Romosozumab lowered the risk of PD development compared with PTHR agonist. The study also highlights the utility of routinely collected health data for drug repositioning. While further validation is warranted, the findings suggest that the Wnt-β-catenin pathway holds promise as a therapeutic target for PD.

## 1 Introduction

Romosozumab, a monoclonal antibody that targets sclerostin, has been approved for the treatment of high-risk osteoporosis (Cosman et al., 2016; Saag et al., 2017). Sclerostin is a physiological inhibitor of the canonical Wnt/β-catenin pathway that blocks Wnt ligand binding to low-density lipoprotein receptor-related protein 5/6, and is primarily produced by osteocytes (Li et al., 2005; Poole et al., 2005). As the Wnt/β-catenin pathway plays an essential role in osteoblast differentiation and function (Li et al., 2005), romosozumab treatment leads to increased bone formation and bone mineral density.

The Wnt/β-catenin pathway plays an essential role in various functions of vast range of tissue cells, such as cell development, homeostasis, fibrogenesis, and stemness (Bastakoty and Young, 2016; Liu et al., 2022). For instance, impairment of this pathway is associated with the pathogenesis of neurodegenerative diseases, such as Alzheimer’s disease and Parkinson’s disease, through the disturbance of neuronal cell homeostasis (Yuan et al., 2019; Marchetti et al., 2020; Schurman et al., 2023) and blood-brain-barrier (BBB) impairment (Ramakrishna et al., 2023). Additionally, Wnt/β-catenin pathway substantially contributes to memory formation of T cells, which is mediated by the transcription factors T cell factor 1 and lymphoid enhancer binding factor 1 (Gattinoni et al., 2009; van Loosdregt and Coffer, 2018). Sclerostin also regulate B cell development in the bone marrow (Sun et al., 2021). While sclerostin is primarily expressed in the bone, it can circulate into the blood (Qureshi et al., 2015); therefore, questions arise regarding whether inhibition of sclerostin and the resulting augmentation of the Wnt/β-catenin pathway have therapeutic effects in other conditions.

Activation of the Wnt/β-catenin pathway has been demonstrated in many cancers, and associated with their invasiveness and stemness (Zhan et al., 2017; Zhang and Wang, 2020). Targeting the Wnt/β-catenin pathway has been recognized as a reasonable molecular target for cancer treatment; however, its dose-limiting toxicity and failure in clinical trials constrain its clinical applicability (Yu et al., 2021). Considering the complexity of the tumor microenvironment, whether activating or inhibiting the Wnt/β-catenin pathway is associated with cancer progression remains an open question. In terms of fibrogenesis, aberrant activation of the Wnt/β-catenin pathway has been observed in the lungs of patients with idiopathic pulmonary fibrosis (Chilosi et al., 2003), and inhibiting this pathway is a potential therapeutic strategy *in vitro* and in preclinical settings (Cao et al., 2018). In contrast, Wnt/β-catenin signaling can also promote protective tissue repair in lung fibrosis, suggesting a complex role of this signaling pathway (Tanjore et al., 2013).

The occurrence of cardiovascular events is currently a concern in romosozumab therapy. In *post hoc* analyses and several meta-analyses of phase 3 trials, a potential increase in cardiovascular events was demonstrated in the romosozumab arm, although the evidence was inconclusive (Lim, 2022). A pharmacovigilance analysis using the United States Food and Drug Administration Adverse Event Reporting System also indicated an increased risk of cardiovascular events with romosozumab (Vestergaard Kvist et al., 2021). In contrast, a recent observational study showed a decreased risk of cardiovascular events compared with a parathyroid hormone receptor (PTHR) agonist (Stokar and Szalat, 2024). Hence, there is insufficient evidence regarding cardiovascular events associated with romosozumab.

Drug repositioning, also known as drug repurposing, aims to identify new therapeutic opportunities for existing drugs that have already undergone extensive safety and efficacy testing (Jourdan et al., 2020). By leveraging the knowledge regarding the mechanisms of action and safety profiles of approved or investigational drugs, drug repurposing offers a cost-effective and time-efficient approach to drug discovery (Hua et al., 2022). Although the effect of romosozumab on osteoporosis is widely accepted, its usefulness for other disorders is not well understood. Moreover, the current knowledge regarding various adverse outcomes is still insufficient. To investigate the influence and potential therapeutic effects of romosozumab for various conditions, an exploratory cohort study was conducted using a nationwide electronic medical record database in Japan. Considering the essential role of Wnt/β-catenin pathway in various processes such as immune function, fibrogenesis, tissue repairing, and neurodegeneration, we have chosen autoimmune diseases, interstitial pneumonia, cardiovascular outcome, Alzheimer’s disease, Parkinson’s disease (PD), serious infections, and malignancies as outcomes of interest.

## 2 Methods

### 2.1 Study design and data source

This retrospective cohort study used the nationwide electronic medical record (EMR) database in Japan, known as the RWD database, which is maintained by the Health, Clinic, and Education Information Evaluation Institute (Kyoto, Japan), with support from JMDC Inc (Tokyo, Japan). The database comprises EMRs, claims data, and diagnosis procedure combination (DPC) data of up to 14 million patients in more than 190 medical institutions as of 2023. The DPC, managed by the Ministry of Health, Labour, and Welfare (Hayashida et al., 2021), is a case-mix classification system for acute inpatient care in Japan and resembles the diagnosis-related group system used in the United States (Wang et al., 2010). The data in the RWD database include diagnoses, medical procedures, prescriptions, hospital admissions, and laboratory data. Data were automatically anonymized and collected in each institution; hence, patients could not be followed-up when they were transferred to another hospital. This study was approved by the Japan Physicians Association Institutional Review Board (approval number: 042-2401-01). Because this study retrospectively utilized an anonymized database, the requirement for informed consent was waived, and an opt-out approach was applied.

### 2.2 Study cohort and drug exposure

Patients were identified according to the following inclusion criteria: (1) those prescribed romosozumab (WHO-Anatomical Therapeutic Chemical (WHO-ATC) code, M05BX06) or PTHR agonists (teriparatide and abaloparatide; WHO-ATC code, H05AA02 and H05AA04), with the first date of prescription designated as Day 0; (2) those with 365 days of continuous enrolment in the database prior to Day 0; (3) those for whom Day 0 was in or after January 2019; (4) those with one or more visits after Day 30; and (5) those aged 18 years or older. Patients prescribed both romosozumab and PTHR agonists on Day 0 were excluded from the study cohort because of ambiguity in the record. PTHR agonists were selected as comparators because they are indicated for severe osteoporosis similar to romosozumab. These drugs are prescribed exclusively by medical institutions in Japan (i.e., they are not available over the counter). All other agents for osteoporosis such as active vitamin D and bisphosphonates can be prescribed regardless of the severity of osteoporosis in Japan, leading to substantial differences in patient backgrounds compared with those prescribed romosozumab. Therefore, agents other than PTHR agonists were not included as comparators. Patients prescribed romosozumab or PTHR agonists on Day 0 were classified into the romosozumab (exposure) and PTHR (comparator) groups, respectively. Drug exposure was considered regardless of drug switching, following intention-to-treat analysis (Gupta, 2011).

### 2.3 Definition of outcomes

The outcomes of interest included autoimmune disease, interstitial pneumonia, cardiovascular outcome, Alzheimer’s disease, PD, serious infections, and malignancies, which were defined according to clinical practice in Japan and previous studies investigating the validity of identifying patients with these diseases. The details are outlined in Supplementary Table S1. For each outcome, patients with a history of the corresponding disease were excluded from the analysis: Day −90 to 0 for acute outcomes (cardiovascular outcome and serious infection) and Day −365 to 0 for other outcomes. As exploratory outcomes, we established three-digit International Classification of Diseases 10th Revision (ICD-10) outcomes (e.g., A01, A02, A03...). Patients with a history of corresponding disease during Day −365 to 0 were excluded from the analysis of the exploratory outcomes.

### 2.4 Covariates

Age, gender, hypertension (ICD-10 code, I10–I15), hyperlipidemia (E78), diabetes (E10–E14), fractures (S02, S12, S22, S32, S42, S52, S62, S72, S82, S92, T02, T08, T10, and T12), components of comorbidities defined in the Charlson Comorbidity Index (Quan et al., 2005), and the frequency of hospital visits (times/month) were used as covariates. Comorbidities were evaluated based on the period from Day −365 to 0, and the frequency of hospital visits was calculated by dividing the number of hospital visits from Day −365 to 0 by 366 and then multiplying by 30.

### 2.5 Statistical analysis

For each outcome, follow-up was started on Day 31 (i.e., 30 days of window period) to consider the latent period and alleviate misidentification of the outcomes that are diagnosed in association with medical management for drug prescription rather than true onset. Therefore, patients who experienced relevant outcomes within 30 days were excluded from the analysis. The patients were followed up until either the last visit date in the database, death date, or the event date, whichever came first. To estimate the adjusted hazard ratio, the stabilized inverse probability-weighted Cox proportional hazard model was employed (Xu et al., 2010). The propensity score for the romosozumab group was calculated using a logistic regression model with covariates and a pseudo-population was established. Variables with a standardized mean difference (SMD) of less than 0.1 were considered well-balanced. Adjusted hazard ratios (HRs), their 95% confidence intervals (CIs), and *p*-value were evaluated for each outcome, and a weighted Kaplan-Meier curve was drawn (Xie and Liu, 2005). The assumption of proportional hazards was tested using Schoenfield’s test. Incidence was also calculated for the pseudo-population. For the exploratory analysis, a volcano plot was depicted with log HR on the x-axis and absolute z-score on the y-axis. Outcomes with fewer than ten observed events were excluded. Taking into account multiplicity, the exploratory outcomes with adjusted *p*-values less than 0.05 in the Benjamini–Hochberg procedure (Benjamini and Hochberg, 1995) were considered potentially influenced by romosozumab treatment.

Subgroup analysis of the outcomes of interest was performed based on age and gender. The age groups were 18–59 years, 60–74 years, and ≥75 years. For *post-hoc* sensitivity analysis, the high-dimensional propensity score (hPS) method was applied for the PD outcome (Schneeweiss et al., 2009). Briefly, diseases based on three-digit ICD-10 codes, drug prescriptions categorized by the fifth level of WHO-ATC codes, and four-digit Japanese category codes of medical procedures were used as variables. The top 200 variables for diseases, drug prescriptions, and medical procedures based on frequency were selected as candidate variables for calculating hPS. These variables were evaluated from Day −365 to Day 0, and those with a frequency of less than 0.01 were omitted. Next, the selected variables were assessed based on their recurrence: ≥1 time, ≥median number of times, and ≥75th percentile number of times. If any of the values were equal, the variable representing the higher cut-off point was excluded. The multiplicative bias term was calculated for each variable (Schneeweiss et al., 2009), and the top 100 variables were chosen as the final variables for the calculation of hPS. Data pre-processing was performed using Python version 3.9.7, and statistical analyses were performed using R version 4.2.3. The study is informed by the reporting of studies conducted using observational routinely collected health data statement for pharmacoepidemiology (RECORD-PE) (Langan et al., 2018).

## 3 Results

### 3.1 Study cohort

**FIGURE 1:**
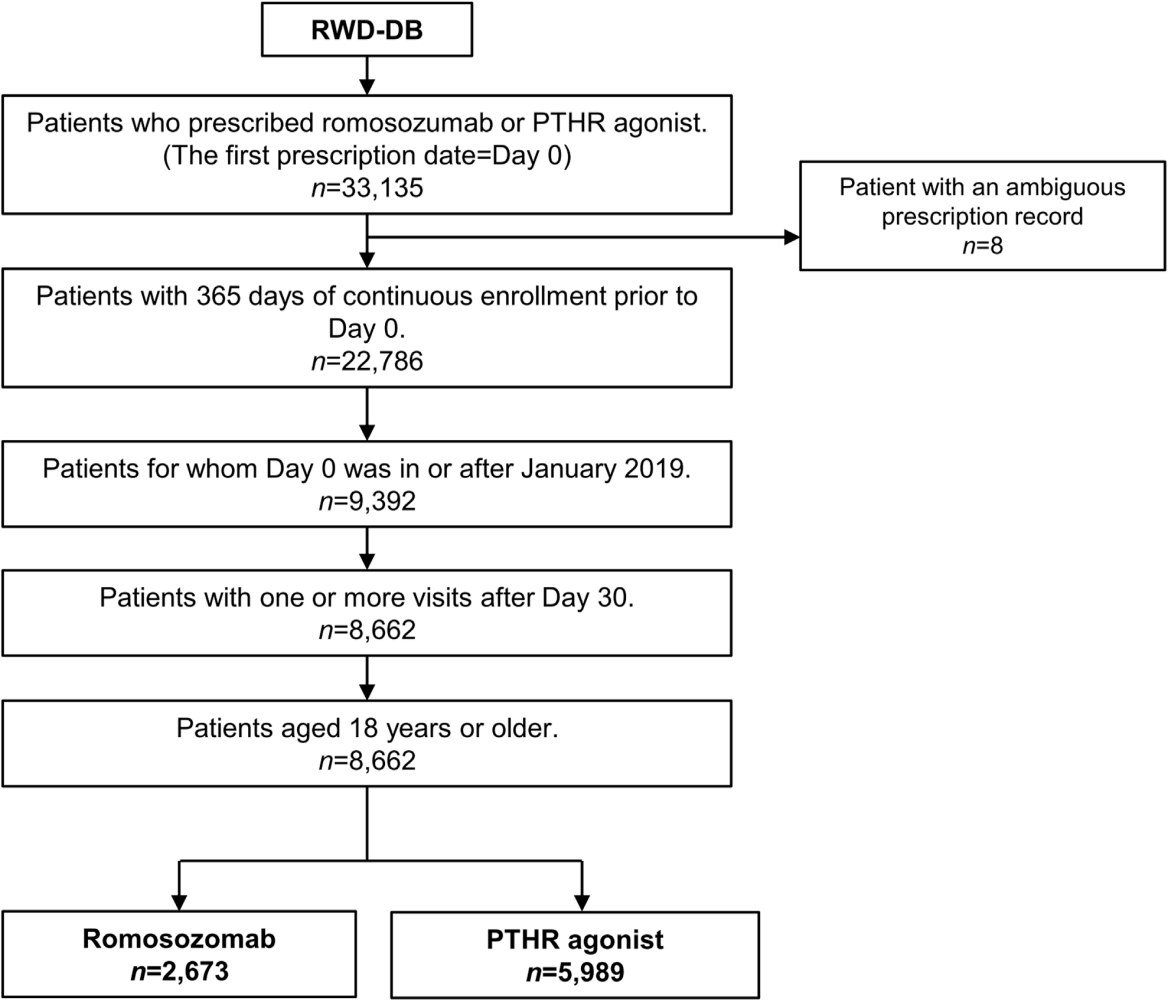
Patient flow. DB, database; PTHR, parathyroid hormone receptor.

In total, 2,673 patients treated with romosozumab and 5,989 patients treated with PTHR agonists were identified (Figure 1). The mean age was 78.3 years (standard deviation [SD], 8.9) in the romosozumab group and 78.6 (SD, 9.5) years in the PTHR agonist group (Table 1). Females comprised the majority of the study cohort, with males accounting for 13.4% of the romosozumab group and 20.1% of the PTHR agonist group. Baseline comorbidities appeared to be imbalanced in several diseases, including myocardial infarction (SMD, 0.124), heart failure (SMD, 0.147), and diabetes (SMD, 0.150). After stabilized inverse probability weighting, all variables showed an SMD of less than 0.1, indicating a well-balanced distribution between the romosozumab and PTHR agonist groups.

**TABLE 1:** Patient characteristics before and after weighting.

Variable	Before weighting			After weighting		
	Romosozumab (*n* = 2,673)	PTHR agonist (*n* = 5,989)	SMD	Romosozumab (*n* = 2,670)	PTHR agonist (*n* = 5,992)	SMD
Age, mean (SD)	78.3 (8.9)	78.6 (9.5)	0.038	78.5 (9.0)	78.5 (9.5)	0.003
Age group, no. (%)			0.056			0.025
18 to 59	85 (3.2)	213 (3.6)		84.8 (3.2)	213.0 (3.6)	
60 to 74	751 (28.1)	1,540 (25.7)		717.4 (26.9)	1,567.6 (26.2)	
75 or older	1,837 (68.7)	4,236 (70.7)		1,867.6 (70.0)	4,211.6 (70.3)	
Male, no. (%)	358 (13.4)	1,206 (20.1)	0.181	491.5 (18.4)	1,082.5 (18.1)	0.009
Comorbidities in CCI, no. (%)						
Myocardial infarction	44 (1.6)	217 (3.6)	0.124	85 (3.2)	180 (3.0)	0.010
Heart failure	436 (16.3)	1,322 (22.1)	0.147	548 (20.5)	1,216 (20.3)	0.006
Peripheral artery diseases	285 (10.7)	721 (12.0)	0.043	309 (11.6)	693 (11.6)	0.001
Cerebrovascular diseases	525 (19.6)	1,407 (23.5)	0.094	598 (22.4)	1,336 (22.3)	0.003
Dimentia	212 (7.9)	570 (9.5)	0.056	236 (8.8)	539 (9.0)	0.006
Chronic pulmonary diseases	435 (16.3)	1,104 (18.4)	0.057	485 (18.2)	1,068 (17.8)	0.009
Rheumatic disease	294 (11.0)	628 (10.5)	0.017	293 (11.0)	642 (10.7)	0.008
Peptic ulcer	606 (22.7)	1,426 (23.8)	0.027	632 (23.7)	1,406 (23.5)	0.005
Mild liver diseases	471 (17.6)	980 (16.4)	0.033	445 (16.7)	1,005 (16.8)	0.002
Diabetes without complications	109 (4.1)	429 (7.2)	0.134	168 (6.3)	372 (6.2)	0.003
Diabetes with complications	151 (5.6)	408 (6.8)	0.048	175 (6.5)	387 (6.5)	0.004
Paraplegia, hemiplegia	40 (1.5)	124 (2.1)	0.043	55 (2.1)	114 (1.9)	0.012
Renal diseases	137 (5.1)	379 (6.3)	0.052	158 (5.9)	357 (6.0)	0.002
Malignancies	594 (22.2)	1,109 (18.5)	0.092	525 (19.7)	1,175 (19.6)	0.001
Moderate-severe liver diseases	14 (0.5)	66 (1.1)	0.064	27 (1.0)	56 (0.9)	0.010
Metastatic malignancies	67 (2.5)	83 (1.4)	0.081	46 (1.7)	104 (1.7)	<0.001
HIV	0 (0.0)	4 (0.1)	0.037	0 (0.0)	3 (0.0)	0.030
Other comorbidities, no. (%)						
Hypertension	1,232 (46.1)	3,150 (52.6)	0.130	1,355 (50.7)	3,032 (50.6)	0.003
Hyperlipidemia	856 (32.0)	2,138 (35.7)	0.078	933 (34.9)	2,077 (34.7)	0.006
Diabetes	774 (29.0)	2,154 (36.0)	0.150	896 (33.5)	2,022 (33.7)	0.004
Fracture	1,988 (74.4)	4,616 (77.1)	0.063	2,051 (76.8)	4,575 (76.4)	0.011
Hospital visits/month, mean (SD)^a^	0.6 (0.4)	0.5 (0.4)	0.157	0.6 (0.4)	0.6 (0.4)	0.018

^a^ Log-transformed.

PTHR, parathyroid hormone receptor; SMD, standardized mean difference; SD, standard deviation; CCI, Charlson Comorbidity Index; HIV, human immunodeficiency virus.

### 3.2 Main analysis

**FIGURE 2:**
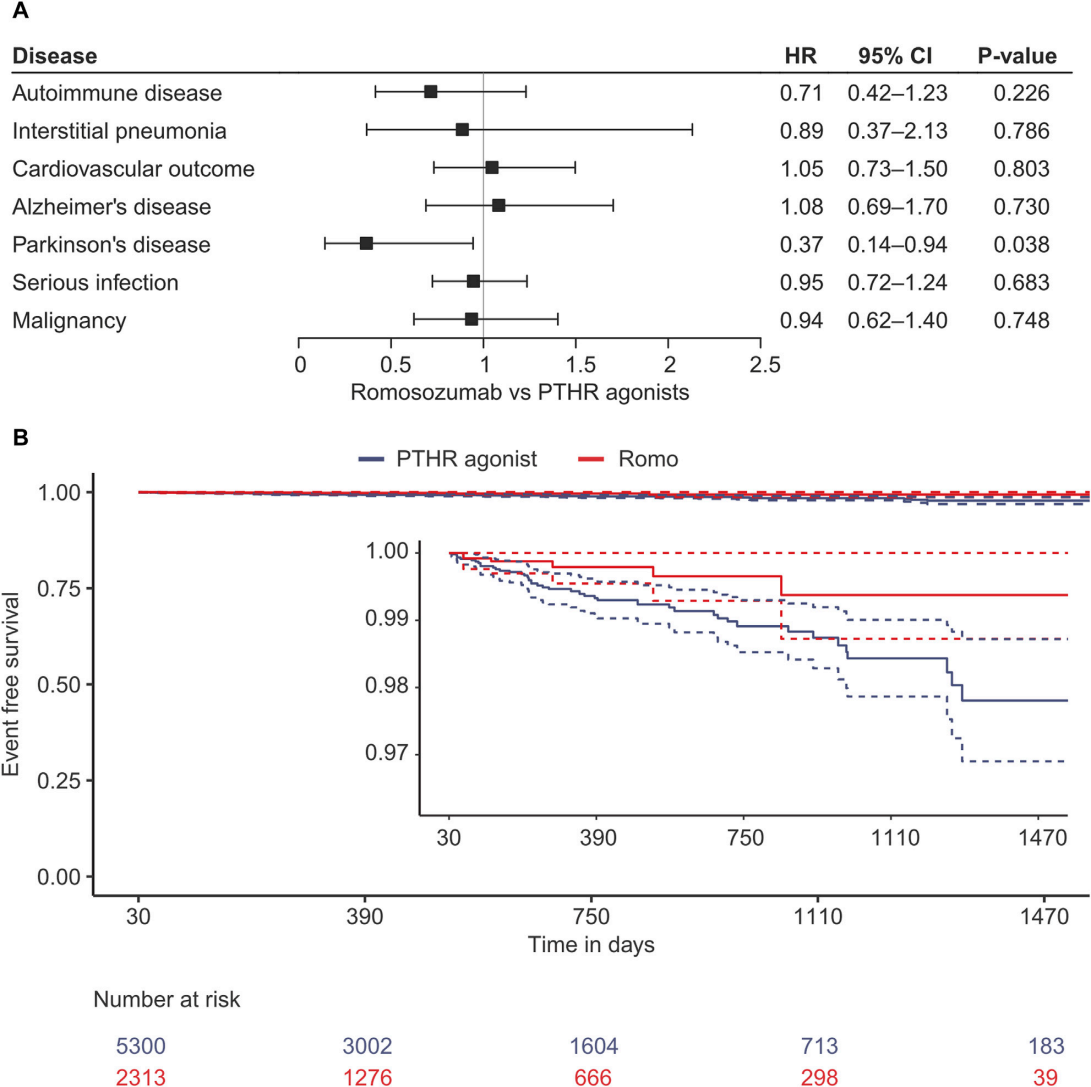
Results of the survival analysis using stabilised inverse probability treatment weighting. **(A)** Forest plot of for each outcome. **(B)** Kaplan-Meier curve for the development of Parkinson’s disease. HR, Hazard ratio; CI, confidence interval; PTH, parathyroid hormone receptor agonist; Romo, romosozumab.

Several outcomes of interest were investigated using stabilized inverse probability-weighted Cox proportional hazard models. Almost all outcomes of interest were not associated with romosozumab use compared to the PTHR agonist, with hazard ratios (HRs) and their 95% confidence intervals (CIs) being 0.71 (0.42–1.23, *p* = 0.226) for autoimmune disease, 0.89 (0.37–2.13, *p* = 0.786) for interstitial pneumonia, 1.05 (0.73–1.50, *p* = 0.803) for the cardiovascular outcome, 1.08 (0.69–1.70, *p* = 0.730) for Alzheimer’s disease, 0.95 (0.72–1.24, *p* = 0.683) for serious infection, and 0.94 (0.62–1.40, *p* = 0.748) for malignancy (Figure 2; Supplementary Table S2). In contrast, the risk of PD decreased in the romosozumab group (0.37 [0.14–0.94], *p* = 0.038) compared with that in PTHR agonist group. To confirm this association, the risk of PD was evaluated using the hPS method as a *post-hoc* sensitivity analysis (Schneeweiss et al., 2009). The estimate yielded a similar trend (HR, 0.66 [0.18–2.38], *p* = 0.528), albeit with a wide confidence interval (Supplementary Table S3).

### 3.3 Subgroup analyses

**FIGURE 3:**
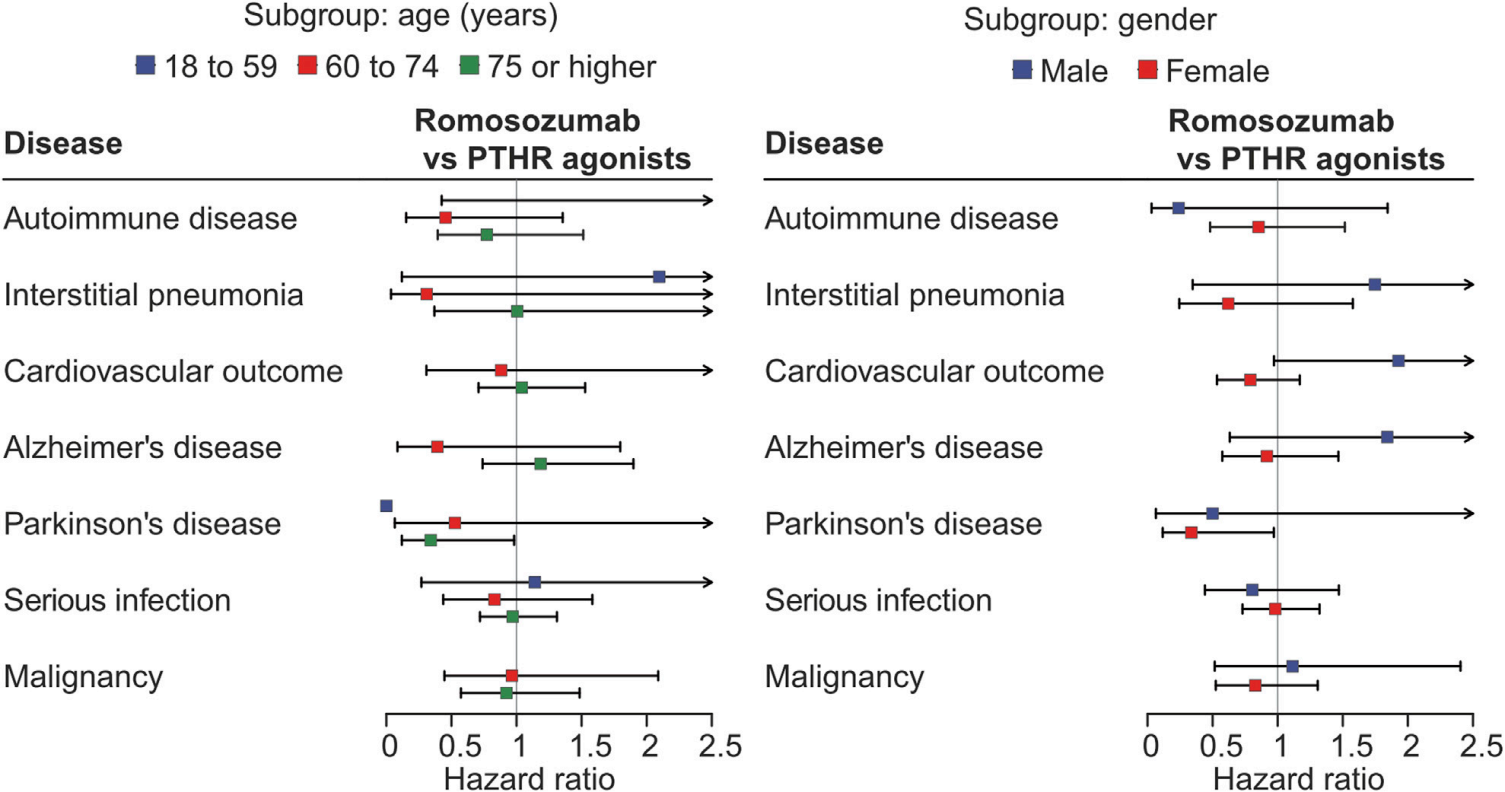
Forest plots for subgroup analysis based on age and gender.

In the subgroup analysis based on age group, the subgroup of 18–59 years showed wide confidence intervals or were not available due to the small sample size and events. The analysis for PD indicated that the protective effect of romosozumab was primarily observed in patients aged ≥75 years; 0.53 (0.06–4.28, *p* = 0.549) for 60–74 years, and 0.34 (0.12–0.98, *p* = 0.046) for ≥75 years. For interstitial pneumonia, the estimate in those aged 60–74 years appeared to be lower than that in those aged ≥75 years (0.31 [0.04–2.67] vs. 1.01 [0.37–2.74], *p* = 0.286 and 0.991, respectively). For Alzheimer’s disease, the HRs were 0.39 (0.09–1.80, *p* = 0.228) for 60–74 years, and 1.18 (0.74–1.90, *p* = 0.482) for ≥75 years. For other outcomes, no remarkable association were observed (Figure 3; Supplementary Table S2).

In the gender-based subgroup analysis, the protective effect against PD was evident in females (0.34 [0.12–0.97] vs. 0.50 [0.06–3.90] for males, *p* = 0.044 and 0.507, respectively). For the cardiovascular outcome, males showed a numerically greater risk than females (1.93 [0.97–3.83] vs 0.79 [0.53–1.17], *p* = 0.060 and *p* = 0.239) (Figure 3; Supplementary Table S2). No apparent associations were observed for the other outcomes.

### 3.4 ICD-10-based exploratory outcomes

The results of the ICD-10-based exploratory analysis are presented in Supplementary Figure S1 and Supplementary Table S4. Among the 903 ICD-10-based outcomes observed, only 16 showed a possible association with romosozumab use. The conditions increased by romosozumab treatment included medical care-associated complications (T88, other complications of surgical and medical care, not elsewhere classified), disease codes associated with insurance claims (M80, osteoporosis with pathological fracture), dental disorders (K05, gingivitis and periodontal diseases; K08, other disorders of teeth and supporting structures), and glomerulonephritis (N05, Unspecified nephritic syndrome). Those decreased by romosozumab treatment included nausea and vomiting (R11), upper respiratory infections (J06, Acute upper respiratory infections of multiple and unspecified sites), sleep disorders (G47), and vestibular disorders (H81, disorders of vestibular function).

## 4 Discussion

This study used a nationwide electronic health record database in Japan to explore conditions that are influenced by romosozumab therapy. As a result, romosozumab has the potential to inhibit the onset of PD. Additionally, while the overall population showed a comparable risk of the cardiovascular outcome compared with PTHR agonists, subgroup analysis suggested a higher risk of the cardiovascular outcome in males. Routinely collected health databases are widely utilized in the field of pharmacoepidemiology to evaluate the real-world effectiveness and safety of drugs (Xu et al., 2010), but this study also demonstrates their utility from the perspective of evaluating roles of off-label drug, namely, drug repositioning (Xie and Liu, 2005).

Romosozumab is a monoclonal antibody against sclerostin, a physiological inhibitor of the Wnt/β-catenin pathway, and this agent promote the effect of the Wnt/β-catenin pathway. The efficacy of romosozumab in the development of PD underscores the importance of the Wnt/β-catenin pathway as a promising molecular target (Schneeweiss et al., 2009; Marchetti et al., 2020). PD is a neurodegenerative disease that has shown an increase in prevalence in parallel with aging in recent years (Abbas et al., 2018; Marchetti et al., 2020), and is characterized by the accumulation of α-synuclein and decline in dopamine levels due to the loss of dopaminergic cells, primarily resulting in motor dysfunction (Stefanis, 2012; Ramakrishna et al., 2023). Abnormal regulation of the Wnt/β-catenin pathway in neural stem cells in the subventricular zones (SVZ) and subgranular zones (SGZ) has been proposed to contribute to the pathogenesis of PD (L’Episcopo et al., 2014; Singh et al., 2018). Impairment of the Wnt/β-catenin pathway has also been implicated in the microglia-astrocyte-neural stem cell crosstalk in aged PD. The M1 phenotype of microglia phenotype is associated with impaired astrocyte proneurogenic ability, which is attributed to impairment of the Wnt/β-catenin pathway in PD model mice (L’Episcopo et al., 2012; L’Episcopo et al., 2013). Furthermore, Wnt activators promote differentiation of dopaminergic neurons (Esfandiari et al., 2012). While various therapeutic targets have been explored in PD, clinical practice has remained limited to interventions targeting dopamine levels, emphasizing the importance of exploring novel treatment approaches, such as the Wnt/β-catenin pathway.

Systemic activation of the Wnt/β-catenin pathway raises concerns regarding off-target effects on healthy tissues and, in some cases, the potential for tumor progression (L’Episcopo et al., 2013). Therefore, it is essential to activate the Wnt/β-catenin pathway in a highly regulated manner to minimize potential side effects. Therapeutic targeting of sclerostin has been considered an attractive therapeutic strategy because the expression of sclerostin is primarily limited to the skeletal tissue (Baron and Rawadi, 2007). Nonetheless, sclerostin has been found not only to exert paracrine effects but also to circulate in the bloodstream (Qureshi et al., 2015). Additionally, disruption of the BBB has been reported in neurodegenerative diseases, such as PD (Esfandiari et al., 2012), indicating that sclerostin can cross the BBB and reach the central nervous system. Thus, the neutralization of circulating sclerostin in blood may exert an impact on the Wnt/β-catenin pathway in the central nervous system. Further investigation of the detailed mechanism of action of romosozumab in the central nervous system is warranted.

It is known that PD is preceded by various non-motor symptoms, such as hyposmia, REM sleep behavior disorder, depression, and constipation, many years before the onset of motor symptoms, a phase known as prodromal PD (Mahlknecht et al., 2015). Since the diagnosis of PD is typically based on motor dysfunction, most diagnoses observed in this study are likely to be at the motor stage of PD rather than the prodromal stage. The Kaplan-Meier curve shows that the risk reduction by romosozumab appears to have already occurred at the time of one to a few years, suggesting that the effect observed in this study may be associated with the progression of the disease from the prodromal stage to the motor stage rather than the initiation of the disease. As mentioned above, romosozumab may improve neuronal homeostasis by enhancing the Wnt/β-catenin pathway, potentially delaying or inhibiting disease progression. However, it remains unclear which stages of PD are modulated by romosozumab.

While the potential efficacy of romosozumab in PD was demonstrated, no clear association was suggested for Alzheimer’s disease in this study. Alzheimer’s disease has also been associated with impairment of the Wnt/β-catenin pathway, as well as PD (Baron and Rawadi, 2007). Recent reports have indicated that serum sclerostin levels are elevated in Alzheimer’s disease in association with high brain amyloid β accumulation (Yuan et al., 2023), and osteocyte-derived sclerostin can traverse the BBB, leading to the accumulation of amyloid β and disease progression in AD model mice (Shi et al., 2024), further supporting the therapeutic potential of the Wnt/β-catenin pathway in Alzheimer’s disease. In this study, both Alzheimer’s and PD were identified not solely by diagnosis records but by more stringent methods (two or more diagnoses, or discharge diagnoses recorded in the DPC data), although the accuracy of this algorithm has not been investigated in the Japanese clinical setting. The possibility that misidentification of these diseases affects the risk estimates cannot be ruled out. Otherwise, the current findings might reflect the differences between Alzheimer’s disease and PD in the pathophysiological roles of the Wnt/β-catenin pathway and sclerostin. Further pathophysiological and epidemiological insights will aid in understanding whether the Wnt/β-catenin pathway and sclerostin could be promising targets in human Alzheimer’s disease.

A null effect of romosozumab on cardiovascular outcomes was observed in the total population; however, a potentially harmful effect was noted in males. This finding aligns with the current safety concerns and those of a previous study (Saag et al., 2017; Fixen and Tunoa, 2021; Vestergaard Kvist et al., 2021; Lim, 2022). The difference between the age groups was not evident. This finding suggests that compared with age, male sex is a key risk factor affecting cardiovascular outcomes among those treated with romosozumab. Currently, patients with a history of cardiovascular disease within the preceding year are contraindicated in the United States (Amgen Inc. Evenitytm, 2019). Unraveling the effect of other known risk factors, such as smoking and obesity, may be helpful in distinguishing patients with a high risk of cardiovascular events from those with a low risk due to romosozumab treatment.

In the analysis of the ICD-10-based exploratory outcomes, only 16 out of 903 outcomes showed a potential association with romosozumab therapy. Medication-related osteonecrosis of the jaw (MRONJ, included in the ICD-10 code K10), which is typically linked to bisphosphonate and denosumab treatments (Yarom et al., 2019), was not detected in this analysis. However, clinical trial data indicate the MRONJ risk ranges from 0.03% to 0.05% (Cosman et al., 2016; Saag et al., 2017), suggesting that the sample size in the study may have been insufficient to detect this signal. In addition, protective effects against upper respiratory tract infections and vestibular disorders were observed. Although caution should be exercised in interpreting the results since these outcomes were defined solely based on diagnosis records, which may result in varying accuracy across different diseases, these findings may serve as valuable references for future research.

This study has several limitations. First, the patients in the RWD database are automatically anonymized within each hospital, which limits the traceability when a patient is transferred to or receives medical management at another hospital. This may lead to underestimation and limited reliability of the outcomes. Second, diagnoses recorded in the database may differ from the actual diagnoses because these records primarily serve insurance claims rather than clinical management by physicians. However, rigorous procedures were employed to identify patients with the outcomes of interest according to previous studies, rather than relying solely on diagnosis records. This approach is considered to have a favorable accuracy for the outcomes of interest. Third, given the relatively low number of PD events, studies utilizing other data sources may be required to validate the findings of the current study. The result of the sensitivity analysis further emphasizes the need for further validation. Fourth, since this study is an active comparator study, the effect of romosozumab might be solely reflect the effect of PTHR agonist with an opposite direction. Fifth, the history of myocardial infarction and heat failure was less frequent in the romosozumab group. This may reflect the clinical practice based on current concerns regarding cardiovascular events. Although the many covariates, including history of cardiovascular diseases and its risk diseases such as diabetes and hypertension, were adjusted for, the possibility of residual confounding and underestimation of the risk in romosozumab group cannot be dismissed. Sixth, we did not account for the possibility of misdiagnosis of other parkinsonism such as vascular parkinsonism and drug-induced parkinsonism. Accurately differentiating these conditions from PD using an EMR database is challenging, and detailed chart review is required to achieve this. Finally, it is important to acknowledge that the database consists of data from restricted medical institutions in Japan. As a result, the database may not necessarily represent the entire population of Japan, thereby limiting its generalizability. Despite these limitations, the current study provides valuable insights owing to the use of a large nationwide EMR database, shedding light on the pathogenesis of PD and the mechanism of action of romosozumab.

In conclusion, this study demonstrated that romosozumab may be effective in preventing the development of PD and provides insights into other outcomes, including cardiovascular events. The utility of routinely collected health data for drug repositioning was also suggested to decipher crucial disease targets. Although further validation through studies using different data sources and pathophysiological experiments is necessary, the findings suggest that Wnt-β-catenin signaling holds promise as a strategy for treating PD.

## Data Availability

The data analyzed in this study is subject to the following licenses/restrictions: The dataset used in this study cannot be shared publicly because the data were obtained from JMDC Inc. The data will be available upon reasonable request to the corresponding author with permission from JMDC Inc. Requests to access these datasets should be directed to inokuchi@rwdata.co.jp.
