# Supplementary material for: Wnt/β-catenin pathway as a potential target for Parkinson’s disease: a cohort study of romosozumab using routinely collected health data in Japan
Source: Front Pharmacol. 2024 Jul 22;15:1411285. doi: 10.3389/fphar.2024.1411285 (PMC11298754; doi:10.3389/fphar.2024.1411285)
Supplement: Supplementary file 1 [file DataSheet1.PDF]

Supplementary Material

1 Supplementary Figures

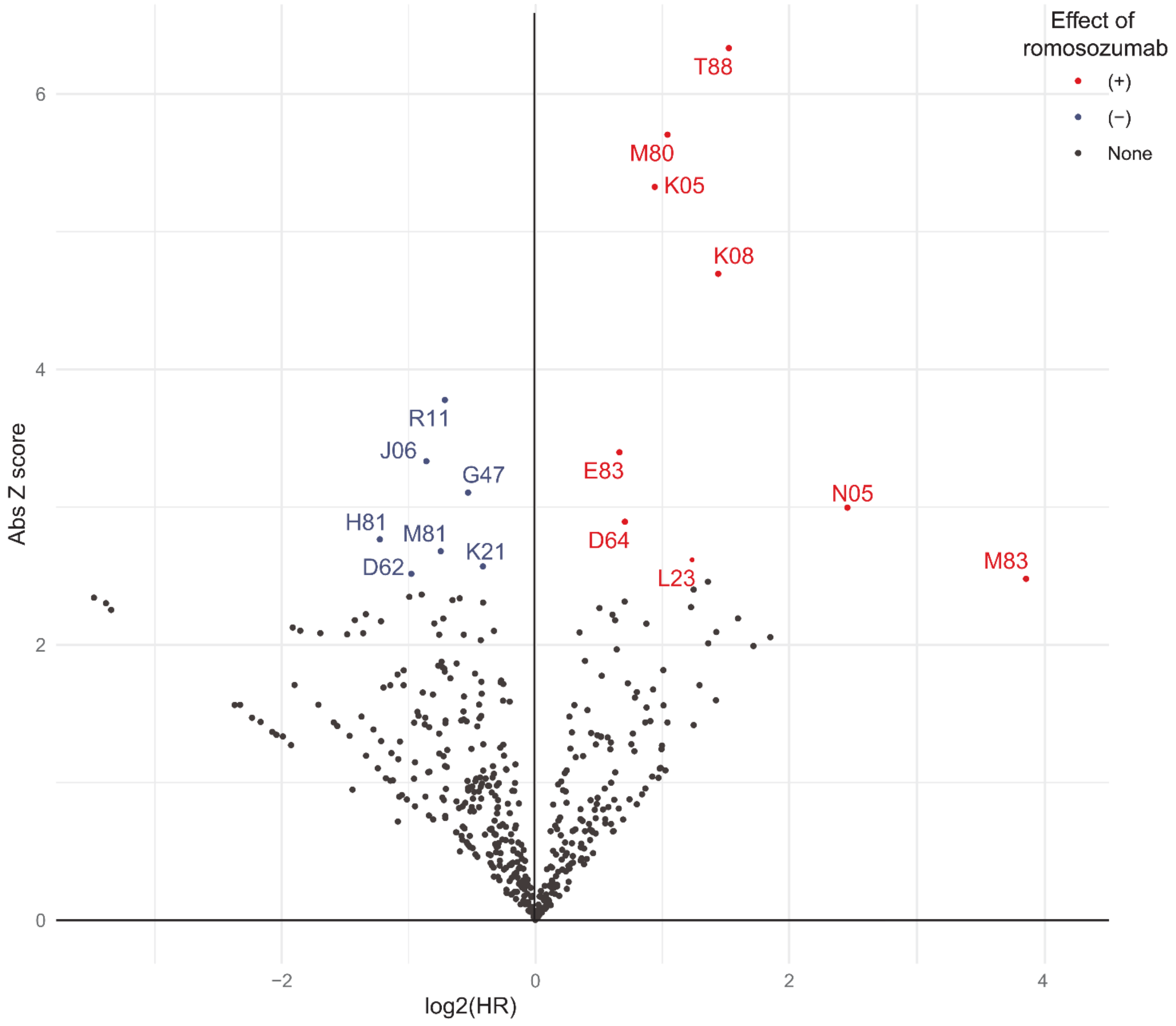

Supplementary Figure S1 | Volcano plot for ICD-10-based exploratory outcomes. A volcano plot was generated for the exploratory outcomes, with the log-transformed adjusted hazard ratio on the x-axis and the absolute z-score on the y-axis. Outcomes fewer than 10 observed events in total were excluded. HR, hazard ratio; Abs, absolute.

2 Supplementary Tables

Supplementary Table S1 | Definition of the outcome of interest

Outcomes of interest	Definition
Autoimmune disease	Autoimmune disease was defined as the composite of 23 diseases. For each disease, we consider the corresponding disease when two or more diagnosis were observed with an interval of 30 to 365 days, referring the study investigating the validity of disease codes for systemic lupus erythematosus, systemic sclerosis, myositis, Sjögren's syndrome, vasculitis, and polymyalgia rheumatic (1).
Interstitial pneumonia	Interstitial pneumonia was identified according to the following steps.<br>a. Two or more diagnosis were observed within an interval of 30 to 365 days (2–4), OR hospital admission due to interstitial pneumonia.<br>b. Computed tomography is performed 90 days before or after step a (2,4,5).
Cardiovascular outcome	Hospital admission due to acute myocardial infarction (6,7), heart failure (6,7) or stroke (7) is defined as the cardiovascular outcome.
Alzheimer's disease, Parkinson's disease	The validity identifying Alzheimer's disease and Parkinson's disease using disease code alone varied across studies (8–11). Taking into account the uncertainty of the disease codes, these outcomes were considered when two or more diagnosis were observed within an interval of 30 to 365 days, OR hospital admission due to the corresponding diseases (12).
Serious infection	Serious infection was defined as hospital admission due to infection. The disease codes were defined according to the previous study (13).
Malignancy	Malignancy were defined as two or more diagnoses were observed within an interval of 30 to 365 days OR hospital admission due to malignancy in combination with chemotherapy drugs (WHO-ATC code: L01), operative procedures (including hematopoietic stem cell transplantation, Japanese category code: K), or radiation therapy (Japanese category code: M000–M004) (14,15).
**Disease code**	**ICD-10 code (Standard disease code)**
Autoimmune diseases	
ANCA-associated vasculitis	M301, M313, M317, M318 (8845513)
Ankylosing spondylitis	M45
Antiphospholipid syndrome	D686 (7100033, 7100034)
Behçet's disease	M352
Crohn’s disease	K50
Dermatomyositis/polymyositis	M33, M360
Hashimoto’s thyroiditis	E063
Graves’ disease	E050
IgG4-related disease	K118 (8848115, 8846061, 5271001), K830 (8848112), K861 (8842274, 8848310), M359 (8848113), N119 (8848114)
Immune thrombocytopenic purpura	D693
Large vessel vasculitis	M314, M315, M316
Mixed connective tissue disease	M351 (7109008, 7109007)
Multiple sclerosis	G35
Neuromyelitis optica spectrum disorder	G360
Other vasculitis	A520, D690, I098, I677, I791, L95, M0520, M303, M310, M318, M319, M364, N017, N082, I776 (8837385, 8838257), M0530 (7148003)

Other rheumatic diseases	M35
Polyarteritis nodosa	M300, M302, M308
Psoriasis	L40
Rheumatoid arthritis	M05, M0600 (8842105, 8833113), M0601–M0609, M068, M069
Sjögren syndrome	M350
Other spondyloarthritis	M02, M074, M075, M076, M468, M469
Systemic lupus erythematosus	M32
Type 1 diabetes	E10
Interstitial pneumonia	J841, J849, M0510 (8840935, 8847737), M321 (8848278), M330 (8848267), M331 (8848302), M351 (8848245)
Acute myocardial infarction	I21
Heart failure	I50
Stroke	I60–I64
Alzheimer's disease	G30
Parkinson's disease	G20
Malignancy	C

WHO-ATC, WHO-Anatomical Therapeutic Chemical; ICD-10, International Classification of Diseases 10th Revision.

Supplementary Table S2 | Results of the outcome of interest

Disease	Romosozumab			PTHR agonist			HR (95% CI)	P value
	N	No. of events	Incidence*	N	No. of events	Incidence*		
**Total population**								
Autoimmune disease	2044	16	57.1	4763	54	78.6	0.71 (0.42–1.23)	0.226
Interstitial pneumonia	2311	10	30.1	5316	26	33.8	0.89 (0.37–2.13)	0.786
Cardiovascular outcome	2276	56	177.4	5283	131	170.6	1.05 (0.73–1.50)	0.803
Alzheimer's disease	2251	35	109.0	5186	79	102.7	1.08 (0.69–1.70)	0.730
Parkinson's disease	2313	7	21.0	5300	44	56.7	0.37 (0.14–0.94)	0.038
Serious infection	2261	82	262.2	5228	207	276.1	0.95 (0.72–1.24)	0.683
Malignancy	2028	38	133.9	4742	98	142.0	0.94 (0.62–1.40)	0.748
**Subgroup: age 18 to 59 years**								
Autoimmune disease	52	2	268.8	151	2	87.0	3.10 (0.43–22.64)	0.264
Interstitial pneumonia	68	1	85.0	186	1	38.9	2.10 (0.12–36.86)	0.613
Cardiovascular outcome	73	1	117.8	189	0	0.0	>100 (>100–>100)	<0.001
Alzheimer's disease	74	0	0.0	192	0	0.0	NA (NA–NA)	NA
Parkinson's disease	73	0	0.0	187	1	36.2	0.00 (0.00–0.00)	<0.001
Serious infection	73	2	218.0	186	5	191.8	1.14 (0.27–4.84)	0.858
Malignancy	67	0	0.0	178	0	0.0	NA (NA–NA)	NA
**Subgroup: age 60 to 74 years**								
Autoimmune disease	532	4	48.9	1182	19	105.8	0.45 (0.15–1.35)	0.157
Interstitial pneumonia	623	1	8.4	1364	6	26.7	0.31 (0.04–2.67)	0.286
Cardiovascular outcome	622	6	62.5	1394	16	71.1	0.88 (0.31–2.52)	0.814
Alzheimer's disease	632	2	16.1	1397	10	44.8	0.39 (0.09–1.80)	0.228
Parkinson's disease	624	2	18.9	1364	8	34.8	0.53 (0.06–4.28)	0.549
Serious infection	620	15	162.7	1379	42	197.5	0.83 (0.44–1.58)	0.575
Malignancy	552	10	123.5	1221	24	126.4	0.97 (0.45–2.09)	0.928
**Subgroup: age 75 or older years**								
Autoimmune disease	1460	10	53.1	3430	33	68.0	0.77 (0.39–1.51)	0.451
Interstitial pneumonia	1620	8	36.5	3765	20	36.3	1.01 (0.37–2.74)	0.991
Cardiovascular outcome	1582	50	229.1	3701	115	221.6	1.04 (0.71–1.53)	0.842
Alzheimer's disease	1546	33	155.2	3596	69	133.4	1.18 (0.74–1.90)	0.482
Parkinson's disease	1616	5	22.9	3750	36	66.6	0.34 (0.12–0.98)	0.046
Serious infection	1568	66	306.2	3663	160	313.5	0.97 (0.72–1.31)	0.847
Malignancy	1408	28	145.1	3344	74	156.4	0.92 (0.57–1.48)	0.739
**Subgroup: gender male**								
Autoimmune disease	379	1	22.5	844	10	96.3	0.24 (0.03–1.84)	0.170
Interstitial pneumonia	423	4	76.2	914	6	45.1	1.75 (0.35–8.80)	0.499
Cardiovascular outcome	416	23	408.4	922	26	213.0	1.93 (0.97–3.83)	0.060
Alzheimer's disease	415	11	182.0	909	12	98.9	1.84 (0.63–5.37)	0.263
Parkinson's disease	416	2	27.2	915	6	51.6	0.50 (0.06–3.90)	0.507
Serious infection	412	19	343.4	905	50	426.6	0.81 (0.44–1.47)	0.481
Malignancy	329	14	311.9	722	27	285.5	1.11 (0.52–2.40)	0.783
**Subgroup: gender female**								
Autoimmune disease	1665	14	64.9	3919	43	75.3	0.85 (0.48–1.52)	0.589
Interstitial pneumonia	1889	5	19.8	4401	21	31.7	0.62 (0.24–1.58)	0.316
Cardiovascular outcome	1860	33	126.9	4361	105	162.6	0.79 (0.53–1.17)	0.239
Alzheimer's disease	1836	24	92.6	4277	67	103.4	0.92 (0.57–1.47)	0.719
Parkinson's disease	1897	5	19.7	4385	38	57.7	0.34 (0.12–0.97)	0.044
Serious infection	1849	63	244.5	4324	157	248.1	0.98 (0.73–1.32)	0.904
Malignancy	1699	24	99.5	4021	72	119.5	0.83 (0.52–1.31)	0.417

PTHR, parathyroid hormone receptor; HR, hazard ratio; CI, confidence interval; NA, not available.
* Unit: 10000-person-years.

Supplementary Table S3 | Results of the sensitivity analyses

Disease	Romosozumab			PTHR agonist			HR (95% CI)	P value
	N	No. of events	Incidence*	N	No. of events	Incidence*		
Parkinson's disease (hPS)	1946	258	35.8	5350	624	53.4	0.66 (0.18–2.38)	0.528

PTHR, parathyroid hormone agonist; HR, hazard ratio; CI, confidence interval; hPS, high-dimensional propensity score.

* Unit: 10000-person-years.

Supplementary Table 4. Results of ICD-10-based exploratory outcomes

	Romosozumab			PTH-analogue						
**ICD-10**	**N**	**no. of events**	**Incidence (/10000-py)**	**N**	**no. of events**	**Incidence (/10000-py)**	**HR (95% CI)**	**Absolute z-score**	**Adjusted p-value**	**Details**
**T88**	2585	79	200.1	5848	65	69.1	2.88 (2.07–3.99)	6.333	<0.001	Other complications of surgical and medical care, not elsewhere classified
**M80**	1645	124	500.8	4520	172	234.3	2.06 (1.61–2.64)	5.704	<0.001	Osteoporosis with pathological fracture
**K05**	2427	126	343.5	5481	154	177.3	1.92 (1.51–2.44)	5.325	<0.001	Gingivitis and periodontal diseases
**K08**	2621	49	121.7	5932	42	44.4	2.71 (1.79–4.12)	4.694	<0.001	Other disorders of teeth and supporting structures
**R11**	2313	76	219.2	5101	280	356.7	0.61 (0.47–0.79)	3.778	<0.001	Nausea and vomiting
**E83**	2548	102	270.0	5808	161	173.9	1.58 (1.21–2.06)	3.398	0.003	Disorders of mineral metabolism
**J06**	2406	40	109.4	5400	167	198.5	0.55 (0.39–0.78)	3.333	0.003	Acute upper respiratory infections of multiple and unspecified sites
**G47**	1860	96	348.7	3944	301	501.8	0.69 (0.55–0.87)	3.105	0.007	Sleep disorders
**N05**	2641	14	33.5	5952	6	6.0	5.50 (1.80–16.77)	2.996	0.01	Unspecified nephritic syndrome
**D64**	2486	72	188.6	5500	104	116.9	1.63 (1.17–2.27)	2.893	0.014	Other anaemias
**H81**	2518	13	33.9	5676	71	78.5	0.43 (0.23–0.78)	2.766	0.021	Disorders of vestibular function
**M81**	386	39	851.1	1157	192	1401.4	0.60 (0.41–0.87)	2.679	0.027	Osteoporosis without pathological fracture
**L23**	2638	21	50.9	5909	20	21.3	2.35 (1.24–4.47)	2.617	0.033	Allergic contact dermatitis
**K21**	1579	117	513.0	3486	347	683.4	0.75 (0.60–0.93)	2.569	0.037	Gastro-oesophageal reflux disease
**D62**	2598	18	45.7	5727	81	88.2	0.51 (0.30–0.86)	2.515	0.043	Acute posthaemorrhagic anaemia
**M83**	2658	6	14.7	5981	1	1.0	14.45 (1.75–119.34)	2.479	0.048	Adult osteomalacia
M20	2633	15	37.2	5908	14	15.1	2.57 (1.21–5.44)	2.458	0.051	Acquired deformities of fingers and toes
M43	2517	16	41.5	5604	15	17.1	2.37 (1.17–4.80)	2.400	0.059	Other deforming dorsopathies
N72	2654	5	13.2	5952	1	1.0	13.01 (1.57–107.88)	2.378	0.063	Inflammatory disease of cervix uteri
M84	2607	24	58.9	5712	97	107.4	0.54 (0.32–0.90)	2.365	0.065	Disorders of continuity of bone
H01	2581	12	30.7	5826	57	61.5	0.50 (0.28–0.89)	2.348	0.067	Other inflammation of eyelid
J93	2660	1	1.5	5972	16	16.7	0.09 (0.01–0.67)	2.342	0.068	Pneumothorax
S00	2497	41	106.4	5607	145	161.9	0.66 (0.47–0.94)	2.338	0.069	Superficial injury of head
R57	2579	33	83.4	5729	121	131.4	0.64 (0.43–0.93)	2.324	0.071	Shock, not elsewhere classified
D63	2613	50	123.6	5844	72	75.7	1.63 (1.08–2.46)	2.313	0.073	Anemia in chronic diseases classified elsewhere
K29	1557	92	412.6	3561	281	545.4	0.75 (0.59–0.96)	2.306	0.074	Gastritis and duodenitis
F50	2647	1	2.5	5951	26	26.6	0.10 (0.01–0.71)	2.302	0.074	Eating disorders
E55	2605	14	34.5	5951	14	14.5	2.34 (1.12–4.87)	2.273	0.08	Vitamin D deficiency

J02	2510	76	201.8	5639	128	143.3	1.42 (1.05–1.92)	2.266	0.081	Acute pharyngitis
D32	2662	6	13.4	5978	2	2.0	6.74 (1.28–35.44)	2.253	0.083	Benign neoplasm of cerebral meninges
G91	2665	1	1.7	5961	17	17.4	0.10 (0.01–0.74)	2.253	0.083	Hydrocephalus
I74	2605	7	17.0	5832	41	43.5	0.40 (0.17–0.90)	2.222	0.09	Arterial embolism and thrombosis
M13	2513	47	122.0	5698	74	80.7	1.52 (1.05–2.21)	2.218	0.09	Other arthritis
M15	2641	10	23.5	5952	8	7.9	3.02 (1.12–8.14)	2.191	0.096	Polyarthrosis
R13	2596	27	67.4	5801	105	112.0	0.60 (0.39–0.95)	2.190	0.096	Dysphagia
K04	2600	44	111.3	5838	68	72.1	1.55 (1.04–2.29)	2.178	0.099	Diseases of pulp and periapical tissues
K55	2644	5	11.6	5939	30	31.1	0.37 (0.15–0.91)	2.179	0.099	Vascular disorders of intestine
K62	2628	8	19.1	5898	43	45.2	0.43 (0.20–0.92)	2.171	0.1	Other diseases of anus and rectum
R31	2569	27	67.6	5783	35	37.0	1.83 (1.06–3.18)	2.153	0.104	Unspecified haematuria
S01	2600	20	50.2	5794	81	86.7	0.58 (0.35–0.95)	2.154	0.104	Open wound of head
T09	2640	3	6.7	5947	24	24.6	0.27 (0.08–0.90)	2.125	0.111	Other injuries of spine and trunk, level unspecified
D52	2658	3	7.1	5942	24	25.2	0.28 (0.08–0.92)	2.102	0.117	Folate deficiency anaemia
L30	2104	131	423.3	4821	382	530.5	0.80 (0.65–0.98)	2.100	0.117	Other dermatitis
R73	2638	13	32.7	5922	12	12.6	2.69 (1.06–6.78)	2.092	0.119	Elevated blood glucose level
M17	1924	121	438.9	4555	232	340.3	1.27 (1.02–1.60)	2.090	0.12	Gonarthrosis [arthrosis of knee]
I34	2615	3	8.0	5832	24	26.1	0.31 (0.10–0.93)	2.084	0.12	Nonrheumatic mitral valve disorders
R18	2659	5	12.0	5965	30	30.7	0.39 (0.16–0.95)	2.084	0.12	Ascites
I51	2597	4	11.1	5851	30	31.7	0.36 (0.14–0.94)	2.076	0.122	Complications and ill-defined descriptions of heart disease
I95	2605	22	54.5	5716	84	91.4	0.59 (0.36–0.97)	2.073	0.122	Hypotension
S72	2167	35	103.9	4865	123	154.3	0.68 (0.47–0.98)	2.073	0.122	Fracture of femur
C67	2654	8	18.3	5947	5	5.0	3.61 (1.06–12.27)	2.055	0.127	Malignant neoplasm of bladder
T81	2458	64	172.7	5372	194	228.3	0.74 (0.56–0.99)	2.033	0.134	Complications of procedures, not elsewhere classified
T15	2646	9	22.9	5964	9	9.5	2.57 (1.02–6.45)	2.010	0.141	Foreign body on external eye
G58	2657	7	17.3	5950	5	5.2	3.29 (1.02–10.63)	1.991	0.147	Other mononeuropathies
H47	2657	4	10.3	5964	1	1.2	8.62 (1.01–73.38)	1.971	0.153	Other disorders of optic [2nd] nerve and visual pathways
L60	2622	34	85.6	5877	52	55.6	1.56 (1.00–2.43)	1.966	0.154	Nail disorders

ICD-10-based outcomes with the absolute z-score >1.96 (95th percentile) are shown. Outcomes less than 10 events observed in total were excluded.
ICD-10, International Classification of Diseases 10th Revision; py, person-years; HR, hazard ratio; CI, confidence interval.

Reference

1. Bernatsky S, Linehan T, Hanly JG. The accuracy of administrative data diagnoses of systemic autoimmune rheumatic diseases. J Rheumatol [Internet]. 2011 Aug;38(8):1612–6. Available from: http://dx.doi.org/10.3899/jrheum.101149

2. Meehan M, Shah A, Lobo J, Oates J, Clinton C, Annapureddy N, et al. Validation of an algorithm to identify incident interstitial lung disease in patients with rheumatoid arthritis. Arthritis Res Ther [Internet]. 2022;24(1):1–11. Available from: http://dx.doi.org/10.1186/s13075-021-02655-z

3. Cho S-K, Doyle TJ, Lee H, Jin Y, Tong AY, Ortiz AJS, et al. Validation of claims-based algorithms to identify interstitial lung disease in patients with rheumatoid arthritis. Semin Arthritis Rheum [Internet]. 2020 Aug;50(4):592–7. Available from: https://linkinghub.elsevier.com/retrieve/pii/S0049017220301025

4. England BR, Roul P, Mahajan TD, Singh N, Yu F, Sayles H, et al. Performance of Administrative Algorithms to Identify Interstitial Lung Disease in Rheumatoid Arthritis. Arthritis Care Res [Internet]. 2020 Oct 29;72(10):1392–403. Available from: https://onlinelibrary.wiley.com/doi/10.1002/acr.24043

5. Morgan A, Gupta RS, George PM, Quint JK. Validation of the recording of idiopathic pulmonary fibrosis in routinely collected electronic healthcare records in England. BMC Pulm Med [Internet]. 2023 Jul 11;23(1):256. Available from: http://dx.doi.org/10.1186/s12890-023-02550-0

6. Nakai M, Iwanaga Y, Sumita Y, Kanaoka K, Kawakami R, Ishii M, et al. Validation of Acute Myocardial Infarction and Heart Failure Diagnoses in Hospitalized Patients With the Nationwide Claim-Based JROAD-DPC Database. Circulation Reports [Internet]. 2021;3(3):131–6. Available from: http://dx.doi.org/10.1253/circrep.cr-21-0004

7. Fujihara K, Yamada-Harada M, Matsubayashi Y, Kitazawa M, Yamamoto M, Yaguchi Y, et al. Accuracy of Japanese claims data in identifying diabetes-related complications. Pharmacoepidemiol Drug Saf [Internet]. 2021;30(5):594–601. Available from: http://dx.doi.org/10.1002/pds.5213

8. McGuinness LA, Warren-Gash C, Moorhouse LR, Thomas SL. The validity of dementia diagnoses in routinely collected electronic health records in the United Kingdom: A systematic review. Pharmacoepidemiol Drug Saf [Internet]. 2019 Feb;28(2):244–55. Available from: http://dx.doi.org/10.1002/pds.4669

9. Wilkinson T, Ly A, Schnier C, Rannikmäe K, Bush K, Brayne C, et al. Identifying dementia cases with routinely collected health data: A systematic review. Alzheimers Dement [Internet]. 2018 Aug;14(8):1038–51. Available from: http://dx.doi.org/10.1016/j.jalz.2018.02.016

10. Harding Z, Wilkinson T, Stevenson A, Horrocks S, Ly A, Schnier C, et al. Identifying Parkinson's disease and parkinsonism cases using routinely collected healthcare data: A systematic review. PLoS One [Internet]. 2019 Jan 31;14(1):e0198736. Available from: http://dx.doi.org/10.1371/journal.pone.0198736

11. Peterson BJ, Rocca WA, Bower JH, Savica R, Mielke MM. Identifying incident Parkinson's disease using administrative diagnostic codes: a validation study. Clin Park Relat Disord [Internet]. 2020 Jun 2;3. Available from: http://dx.doi.org/10.1016/j.prdoa.2020.100061

12. Yamana H, Moriwaki M, Horiguchi H, Kodan M, Fushimi K, Yasunaga H. Validity of diagnoses, procedures, and laboratory data in Japanese administrative data. Journal of Epidemiology [Internet]. 2017;27(10):476–82. Available from: http://dx.doi.org/10.1016/j.je.2016.09.009

13. Pawar A, Desai RJ, Solomon DH, Santiago Ortiz AJ, Gale S, Bao M, et al. Risk of serious infections in tocilizumab versus other biologic drugs in patients with rheumatoid arthritis: A multidatabase cohort study. Ann Rheum Dis [Internet]. 2019;78(4):456–64. Available from: http://dx.doi.org/10.1136/annrheumdis-2018-214367

14. Setoguchi S, Solomon DH, Glynn RJ, Cook EF, Levin R, Schneeweiss S. Agreement of diagnosis and its date for hematologic malignancies and solid tumors between medicare claims and cancer registry data. Cancer Causes Control [Internet]. 2007 Jun;18(5):561–9. Available from: http://dx.doi.org/10.1007/s10552-007-0131-1

15. Nishikawa A, Yoshinaga E, Nakamura M, Suzuki M, Kido K, Tsujimoto N, et al. Validation Study of Algorithms to Identify Malignant Tumors and Serious Infections in a Japanese Administrative Healthcare Database. Annals of Clinical Epidemiology [Internet]. 2022;4(1):22004. Available from: https://www.jstage.jst.go.jp/article/ace/4/1/4_22004/_article
